# Herbal and nano-based herbal medicine: New insights into their therapeutic aspects against periodontitis

**DOI:** 10.22038/AJP.2023.23261

**Published:** 2024

**Authors:** Fatemeh Rezaei-Tazangi, Armita Forutan Mirhosseini, Amirhossein Fathi, Hossein Roghani-Shahraki, Reza Arefnezhad, Fateme Vasei

**Affiliations:** 1 *Department of Anatomy, School of Medicine, Fasa Univerity of Medical Sciences, Fasa, Iran*; 2 *Student Research Committee, School of Dentistry, Kerman University of Medical Sciences, Kerman, Iran*; 3 *Department of Prosthodontics, Dental Materials Research Center, Dental Research Institute, School of Dentistry, Isfahan University of Medical Sciences, Isfahan, Iran*; 4 *Student Research Committee, Shiraz University of Medical Sciences, Shiraz, Iran*; 5 *Department of Anatomy, School of Medicine, Shiraz University of Medical Sciences, Shiraz, Iran*; 6 *School of Dentistry, Shiraz University of Medical Sciences, Shiraz, Iran*

**Keywords:** Periodontitis, Herbal therapy, Nano-based formulations

## Abstract

**Objective:**

Periodontitis is a type of prevalent chronic inflammatory disorder resulting in a failure in the function of tissues supporting the tooth, like gingiva, alveolar bone, and periodontal ligament. Although antibiotic therapy is a common therapy for periodontitis cases, this approach can cause some adverse effects in these patients. Thus, finding an effective curative option with low side effects is still a puzzle.

**Materials and Methods:**

This narrative review was conducted on the effects of herbal and nano-based herbal medicine against periodontitis by searching different databases such as Google Scholar, PubMed, Scopus, Web of Science, Science Direct, and Scientific Information Databases.

**Results:**

According to published studies, some popular herbal formulations, such as Aloe vera, curcumin, Melaleuca alternifolia, and Scutellaria baicalensis Georgi, can be effective in periodontitis treatment. However, these herbal products may be accompanied by some pharmacological limitations, such as poor bioavailability, instability, and weak water solubility. On the other hand, harnessing nano-based herbal formulations can elevate the bioavailability, diminish toxicity, and omit repeated administration of drugs.

**Conclusion:**

Herbal and nano-based herbal products can create a good chance to treat periodontitis efficiently.

## Introduction

Periodontitis is categorized as one of the chronic inflammatory diseases causing the impairment of the integrity of tooth-supporting tissues, such as gingiva, alveolar bone, and periodontal ligament, collectively known as the periodontium (ArefNezhad et al., 2022 ▶; Hajishengallis, 2015 ▶). It is reported that periodontitis in severe form has a prevalence of 11.2% globally (Nilsson, 2018 ▶). This oral problem has a wide variety of manifestations, like bleeding during brushing or flossing. Also, tenderness and pain during chewing of specific substances, receding gums, sensitive teeth, the production of discoloring plaque, tooth mobility, and the loss of teeth are the more severe symptoms noted in periodontal diseases (Gasner and Schure, 2021 ▶). Many risk factors have been mentioned for disease development, especially diabetes mellitus, smoking, and poor oral hygiene (Lertpimonchai et al., 2017 ▶). Periodontitis has also been associated with some systemic conditions, such as diabetes, respiratory disorders, chronic renal disease, metabolic syndrome, and cardiovascular diseases (Craig, 2008 ▶; Irani et al., 2015 ▶; Preshaw and Bissett, 2019 ▶; Suzuki et al., 2010 ▶). Moreover, some oral anaerobic bacteria, including *Treponema denticola*, *Porphyromonas gingivalis*, and *Tannerella forsythia*, have a causative role in this disease (Socransky and Haffajee, 2005 ▶). Presently, the common therapeutic approach for periodontitis treatment is intra-pocket-targeted delivery systems of antibiotics in dental pharmacotherapy (Jain et al., 2008 ▶). However, It is associated with the risk of nephritis, allergy, gastrointestinal and hematological disorders, and nervous system impairment in cases with periodontal disorders who received this therapy (Heta and Robo, 2018 ▶). Fortunately, herbal therapy, as complementary and alternative medicine, is considered an effective remedy for improving different diseases from ancient to the present time (Samadi et al., 2022 ▶; Rezaee-Tazangi et al., 2020 ▶). In this field, some popular herbal products, like Aloe vera, curcumin, Scutellaria baicalensis Georgi, and Melaleuca alternifolia have provided a promising outlook for the amelioration of this oral condition (Akbik et al., 2014 ▶; Bhat et al., 2018 ▶; Forouzanfar, 2020 ▶; Tankeu, 2014 ▶; Yang et al., 2012 ▶; Zanuzzo et al., 2017 ▶). Furthermore, reports showed that some nanotechnology-based drug delivery systems, e.g. nanoparticles (NPs), liposomes, nanomicelles, branched dendrimers, and nanocapsules have good potential in medicine (Rezaei-Tazangi et al., 2021 ▶; Suri et al., 2007 ▶). Interestingly, it has been declared that herbal formulations formed on the basis of nanotechnology have a higher ability in treating various disorders (Barkat et al., 2020 ▶). These nano-based herbal formulations can also overcome pharmacological obstacles of herbal medicine, like weak water solubility and bioavailability, and instability (Rezaei-Tazangi et al., 2021 ▶). This is the first study in which the efficiency of some popular herbal and nano-based herbal products on periodontitis through a mechanistic insight was discussed.

## Materials and Methods

In this review study, we gathered accessible data from Google Scholar, PubMed, Scopus, Web of Science, Science Direct, and Scientific Information Databases until 2022. The MeSH terms and free keywords used in this study were: periodontitis, natural products, herbal medicine, herbal extract, nano, nano-based herbal therapy, nano-based herbal medicine, nanotechnology, nano-based herbal formulations, *aloe vera*, *curcumin, melaleuca alternifolia, Scutellaria baicalensis Georgi*, *in vitro*, *in vivo*, animal model, clinical, clinical trial, and clinical study. According to the search strategy, 138 articles were found.  After checking the titles and abstracts, 97 relevant papers were evaluated. The assessed papers were about herbal medicine and nano-based herbal formulations against periodontitis. The figures included in this study were created by the web-based software BioRender. 

## Results

**Periodontitis and its pathogenesis**

**Figure1:**
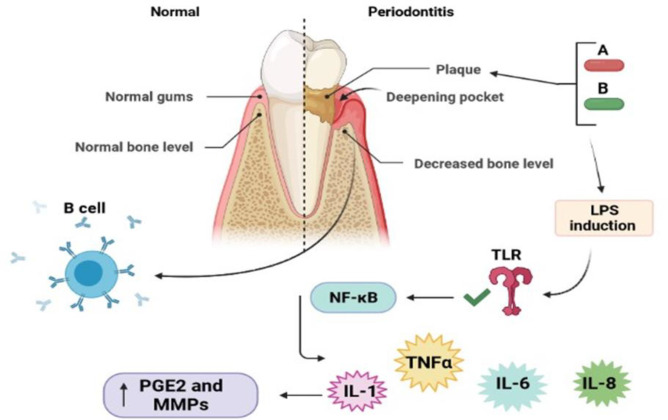
The role of the immune system and bacterial pathogens in pathogenic mechanisms of periodontitis. A, *P. gingivalis*; B, A, *actinomycetemcomitans*; LPS, Lipopolysaccharide; TLR, Toll-like receptors; NF-kB, Nuclear factor-kappa B; TNF-α, tumors necrosis factor-α; PGE2, prostaglandin E2; MMPs, matrix metalloproteinases; interleukin 1; IL-6, interleukin 6; IL-8, interleukin 8

The adaptive and innate immune systems are involved and work together in the pathogenesis of periodontitis (Sell et al., 2017 ▶; Zacarias et al., 2019 ▶). Regarding the adaptive immune system, decreased responses of Th1 cells and increased responses of Th2 cells have been expressed (Seymour et al., 1993 ▶; Sigusch et al., 1998 ▶). In this system, interleukin (IL)-1 has a key role in the destruction of periodontal tissue and may mediate collagenolytic induction and bone-destruction factors, such as prostaglandin E2 (PGE2) and matrix metalloproteinases (MMPs) (Figure 1) (Bascones Martínez et al., 2009 ▶; Mariano et al., 2010 ▶). The innate immune reaction is performed in the disease by phagocytes (e.g. natural killer cells, neutrophils, and dendritic cells). These innate immune cells can be recruited into the infection site as a result of elevated levels of cytokines, such as interferon (IFN)-γ, IL-1β, IL-4, and IL-6 (Cairo et al., 2010 ▶; Meyle et al., 2017 ▶; Ramadan et al., 2020 ▶). Natural killer cells may participate in the resorption of alveolar bone and systemic inflammation in reaction to oral infections (Aoki‐Nonaka et al., 2014 ▶). Another involved agent in the disease is neutrophils producing reactive oxygen species (ROS) (Hirschfeld, 2020 ▶; Scott and Krauss, 2012). An imbalance between the anti-oxidative protection and ROS production in periodontitis pathogenesis has been demonstrated. Increased ROS levels can trigger intracellular signals related to autophagy, which has a dual role in the disease by enhancing cell death or suppressing apoptosis in infected tissues (Liu et al., 2017 ▶). Notably, the increment of neutrophil ROS formation is linked with the increased neutrophil extracellular trap (NET) secretion leading to neutrophil recruitment and tissue damage (Kolaparthy et al., 2014 ▶; Mayadas et al., 2009 ▶). A number of Gram-negative bacteria, for instance, *Porphyromonas* *gingivalis* and *A* *ggregatibacter actinomycetemcomitans*, can also form subgingival plaques causing periodontitis progression (Gölz et al., 2014 ▶). These bacteria, through lipopolysaccharides (LPSs) present in their cell walls, trigger Toll-like receptors (TLR), which in turn activate nuclear factor ĸB (NF-ĸB). As a result, some inflammatory cytokines and chemokines are secreted, for example, IL-1, tumor necrosis factor (TNF)-α, and IL-6 (Kagiya, 2016 ▶; Venugopal et al., 2018 ▶). Recent reports revealed that *P. gingivalis* can change adaptive immune responses. Particularly, *P. gingivalis* interaction with dendritic cells provokes a cytokine pattern that has a helping role in the polarization of T helper (Th) 17 cells. Furthermore, *P. gingivalis* suppresses the formation of gingival epithelial cell-related cytokines recruiting Th1 cells (Hajishengallis, 2014 ▶; Olsen et al., 2016 ▶; Wilensky et al., 2015 ▶). Periodontal epithelium creates a physical obstacle against infection and has a fundamental role in the host innate immune system (Mariano et al., 2010 ▶). In terms of genetic aspects, it is approved that long non-coding RNAs (lncRNAs) have a substantial role in periodontitis development. Also, dysregulation of these transcripts, such as ANRIL, UCA1, FGD5-AS1, FAS-AS1, NEAT1, NKILA, Linc-RAM, and FAS-AS1, in blood samples or gingival tissues of periodontitis cases compared with normal subjects has been addressed (Sayad et al., 2020 ▶). Besides, many single nucleotide polymorphisms (SNPs) located in ANRIL, for instance, rs1333048, rs1333049, rs496892, and rs7865618, have been related to periodontitis risk in diverse populations (Motterle et al., 2012 ▶). 

**Herbal and nano-based herbal therapy: An opportunity for therapeutic purposes or not?**

Herbal therapy is a common and popular therapy for treating many disorders in many areas, such as India, China, and Indonesia, since the ancient era due to its advantages compared with synthetic drugs, like a lower rate of drug reactions and being safe and gentle (Ilyas, 2020 ▶; Khogta et al., 2020 ▶). On the contrary, some side effects have been reported for herbal medicine, like the possibility of overdose potential of herbal drugs. Also, the use of this remedy can cause many cutaneous reactions (Bedi and Shenefelt, 2002 ▶). On the other hand, nano-based drug delivery systems have many benefits, like biocompatibility improvement, modifiable release profiles, and nanoscale size (Majidzadeh et al., 2020 ▶). The utilization of these systems (e.g. NPs, liposomes, ethosomes, phytosomes, solid-lipid NPs, transferosomes, microsphere, and microemulsion/ nanoemulsion) for herbal products may reduce the repeated administration, overcome non-compliance, increase the therapeutic value, reduce toxicity, and increase the bioavailability (Chaudhari and Randive, 2020 ▶; Mamillapalli, 2016 ▶). Some other advantages of nano-based herbal therapy are enhancement of solubility, potentiation of pharmacological activity and stability, improvement of the distribution of tissue macrophages, sustained delivery, and protection from physical and chemical degradation (Mamillapalli, 2016 ▶). Thus, the nano-based herbal formulation may create a large chance to promote the effectiveness of herbal therapy.

***Aloe vera*** **and its nanoformulations: Their effects on periodontitis treatment**

*Aloe vera* is a member of the Liliaceae family and is used in many countries for different therapeutic purposes, like treating diabetes, cardiovascular diseases, and metabolic syndrome (Choudhary et al., 2014 ▶; Guo and Mei, 2016 ▶; Sabbaghzadegan et al., 2021 ▶; Sahu et al., 2013 ▶; Shakib et al., 2019 ▶). This plant in light of its active components, like polysaccharides, anthraquinones, and glycoproteins, can possess many therapeutic effects, such as antiviral, anti-cancer, and anti-ulcer effects (Choi and Chung, 2003 ▶; Gao et al., 2019 ▶). *Aloe vera* can also exert ameliorative effects on supporting tissues of the tooth (e.g. periodontal ligament) and oral conditions like periodontitis. For example, an *in vitro* work revealed that exposing periodontal ligament cells to *Aloe vera* gel can give rise to the preservation of periodontal ligament cell viability (Fulzele et al., 2016 ▶). An *in vivo* study also investigated the influences of the administration of *Aloe vera* hydrogel topically (1 min) on the population of neutrophil cells in animal models of aggressive periodontitis induced by *A. actinomycetemcomitans*. Finally, they declared that using this hydrogel in the concentration of 2.5%, 5%, 10%, and 20% can significantly decrease the number of neutrophil cells, as inflammatory factors that are able to phagocyte bacteria infiltrating the tissue of gingiva (Table 1) (Prasetya et al., 2014 ▶; Susanto et al., 2021 ▶).

**Table 1:** List of studies in which the effect of Aloe vera formulations on periodontitis has been investigated

**Author/ year**	***In vivo*** **/** ***in vitro*** **/ human**	**Herbal / others**	**Effect/mechanism**
	Human	*Aloe vera* gel	Decrease of plaque, pocket depth, and gingival indices
Ashouri Moghaddam et al. 2017	Human	*Aloe vera* gel	Decrease of plaque index
	Human	*Aloe vera* gel	Decrease of the activity of *P. intermedia *and *P. gingivalis* bacteria
	*In vivo*	*Aloe vera* gel	Reduction of inflammatory reactions and caspase-3 area
	Human	*Aloe vera* gel	Decrease of pocket depth index and gingival inflammation
	Human	*Aloe vera* extract	Wound healing effects following periodontal flap surgery
	Human	*Aloe vera* extract	Wound healing effects following periodontal flap surgery
	Human	*Aloe vera* extract	Reduction of plaque and gingival indices
	Human	*Aloe vera* gel	Reduction of plaque, bleeding, and pocket depth indices
	Human	*Aloe vera* gel	Decrease of pocket depth, gingival, and bleeding indices
	Human	*Aloe vera* gel	Decrease of plaque, gingival, bleeding, and pocket depth indices
	*In vivo*	*Aloe vera* hydrogel	Reduction of the number of neutrophil cells

 Hydrogels are biomaterials like extracellular matrix (ECM) in terms of porous structures and have high biocompatibility; therefore, they can be useful for carrying drugs to cells (Buwalda et al., 2017 ▶). Other therapeutic influences of *Aloe vera* comprise anti-bacterial, anti-oxidative, and anti-inflammatory impacts (Langmead et al., 2004 ▶; Nejatzadeh-Barandozi, 2013 ▶). *Aloe vera* consumption exerts its anti-bacterial effect on *Staphylococcus aureus, Streptococcus pyogenes, Klebsiella pneumoniae, Pseudomonas aeruginosa, Propionibacterium acne, Escherichia coli, Salmonella typhi, Helicobacter pylori*, Streptococcus* mutans*, and Streptococcus* sanguis*. Among these bacteria, *E. coli, K. pneumoniae, P. aeruginosa, and S. aureus *are found in periodontitis patients; thus, they can be affected by the anti-bacterial effect of *Aloe vera* causing reduction of plaque and improving the periodontal health (Lawrence et al., 2009 ▶; Penmetsa et al., 2019 ▶; Souto et al., 2006 ▶). Also, the antioxidant properties of *Aloe vera* have been reported by Aggarwal et al. by suppressing the formation of free oxygen radicals through the activated polymorphonuclear leukocytes (Aggarwal et al., 2011 ▶). One of the antioxidant agents present in *Aloe vera* is vitamin C, which has a role in collagen synthesis and increases oxygen levels in the wound region through blood vessel dilation (Figure 3) (Hudwekar et al., 2019 ▶; Wang et al., 2017 ▶). Another possible mechanism of this herb in periodontitis therapy is the inhibition of stimulated granulocyte MMPs, which gives rise to the inhibition of cyclooxygenase (COX) and lipoxygenase (LOX) pathways (Bhat et al., 2011 ▶). The suppression of the COX pathway and reduction of prostaglandin synthesis are among the mechanisms of *Aloe vera *to inhibit inflammation (Vangipuram et al., 2016 ▶). Also, some clinical evaluations indicated the positive effects of this plant against periodontitis cases (Adam et al., 2018 ▶; Hudwekar et al., 2019 ▶; Karim et al., 2014 ▶). In a randomized controlled trial, the effectiveness of mouthwash with *Aloe vera* juice (0.001% Spearmint flavor, 0.2% preservative, 99% aloe juice, and sorbitol for sweetening) on gingival inflammation and plaque accumulation was assessed, and it was shown that it can be an alternative way to treat and prevent gingivitis by reducing plaque and gingival indices (Vangipuram et al., 2016 ▶). Moreover, subgingival administration of the gel form of *Aloe vera* in periodontal pockets of periodontitis subjects ameliorated periodontal disorder by improving clinical parameters, like gingival, plaque, and pocket depth indices (Bhat et al., 2011 ▶). However, the toxic and carcinogenic impacts of this plant have been stated in some papers (Guo and Mei, 2016 ▶). Some evidence addressed the possible therapeutic capacity of nano-based formulations of this herb against the disease. In this direction, Subramani et al. explored the anti-bacterial features of herbal NPs obtained from the shade‐dried gel of *Aloe vera *(Subramani et al., 2018 ▶). In this experiment, The NPs were combined with chitosan polymer and subsequently were coated on cotton fabrics. At the end of the study, they concluded that these chitosan nanocomposites have anti-bacterial effects against *E. coli* and *S. aureus*, which are related to the disease induction and progression, respectively (Gürkan et al., 2009 ▶; Passariello et al., 2012 ▶; Subramani et al., 2018 ▶). Chitosan biomaterials have special characteristics, such as biodegradability, biocompatibility, muco-adhesion, and non-toxicity. Plus, chitosan is the sole cationic polysaccharide in the world with the ability of modification to its derivatives chemically (Fakhri et al., 2020 ▶). In another work, the possible bactericidal effects of silver NPs synthesized by *Aloe vera* and neem on dental pathogens resulting in dental caries and periodontitis, comprising *Enterococcus faecalis*, *S. aureus*, *S. mutans*, and *Pseudomonas* species, were studied using the agar well diffusion method (Rajeshkumar et al., 2019 ▶). At the end of the research, they demonstrated the anti-bacterial effects of these silver NPs against *Pseudomonas* species and *S. mutans* (Rajeshkumar et al., 2019 ▶). Silver NPs incorporated into biomaterials have the capability to diminish or prevent biofilm creation, and they have a considerable antimicrobial function due to their small particle size and large surface-to-volume ratio (Bapat et al., 2018 ▶). Therefore, it seems that these nano-based drug delivery systems, like NPs combined with chitosan polymer and silver NPs, may promote the curative and pharmacological effects of *Aloe vera *on periodontitis mainly through inhibitory influences on dental biofilm and dental pathogens. 

**Curcumin** **and its nanoformulations: Their effects on periodontitis treatment**

Curcumin is derived from the underground stem or the rhizome of a ginger-like plant from the *Zingiberaceae* (ginger) family and contains several active components, including curcuminoids, triterpenoids, diterpenes, and sesquiterpenes (Catanzaro et al., 2018 ▶; Lal, 2012 ▶). This polyphenol possesses many pharmacological influences, like anti-inflammatory, anti-oxidative, and anti-cancer properties (Damiano et al., 2021 ▶; Sharma et al., 2005 ▶). Curcumin consumption can contribute to the management of many complicated conditions, for instance, cardiovascular disorders, metabolic syndrome, arthritis, and anxiety (Hewlings and Kalman, 2017 ▶; Pourbagher-Shahri et al., 2021 ▶). Reports have also approved the therapeutic potential of curcumin against some oral problems, such as periodontitis (Al‐Maweri et al., 2022 ▶; Iova et al., 2021 ▶; Li et al., 2021 ▶). In this regard, documents indicate that curcumin improves osteogenic differentiation, elevates cell proliferation, and decreases the apoptosis and ROS levels of periodontal ligament stem cells by different mechanisms, like affecting the PI3K/AKT/Nrf2 signaling pathway and early growth response gene 1 (EGR1) expression (Figure 3) ( Shi et al., 2021 ▶; Tan et al., 2021 ▶; Xiong et al., 2020 ▶). Bhatia and co-workers addressed the anti-bacterial effects of this plant-derived agent on *P. gingivalis*, *Fusobacterium nucleatum*, *Capnocytophaga*, and Prevotella* intermedia* and its therapeutic activities in chronic periodontitis patients by promoting clinical parameters, for example, plaque, bleeding, and clinical attachment indices (Table 2) (Bhatia et al., 2014 ▶). Indeed, they inserted 1% curcumin gel locally into periodontal pockets, and pluronic 407 (PF-127) hydrogel was utilized as a local drug delivery system in this work (Bhatia et al., 2014 ▶). The hydrogel of Pluronic F-127, a nonionic surfactant, has several advantages, such as non- immunogenicity, non-toxicity, prolonged drug release, and thermo reversibility (Álvarez et al., 2011 ▶). 

**Table 2:** Curcumin in different formulations can target periodontitis efficiently

**Author/ year**	***In vivo*** **/** ***in vitro*** **/ human**	**Herbal/nano-based herbal/ others**	**Effect/mechanism**
	*In vivo*	Curcumin	Suppression of cytokine gene expression, elevation of fibroblastic cell number and collagen content, and decrease of infiltration of inflammatory cells
	*In vivo*/*in vitro*	Curcumin	Decrease of gingival inflammation, alveolar bone loss, and TNF-α and IL-1β formation, regulation of collagen fibers, and suppression of NF-κB activation
	*In vivo*	Curcumin	Decrease of inflammatory cell infiltration and numbers of osteoclasts, apoptotic cells, and osteocytes
	*In vivo*	Curcumin	Reduction of alveolar bone loss and IL-1β and INF-γ levels
	*In vivo*	Curcumin	Reduction of NF-ĸB triggering and promotion of collagen repair and TGF-β level
	*In vivo*	Curcumin	Reduction of oxidative stress
	*In vivo*	Curcumin	Reduction of alveolar bone loss and TNF-α, INF-γ, IL-1β, and IL-6 levels
	*In vivo*	Curcumin	Suppression of cytokine gene expression and NF-κB activation, decrease of inflammatory cell infiltration, and elevation of collagen content and the number of fibroblastic cells
	*In vivo *	Curcumin	Reduction of alveolar bone loss, receptor activator of nuclear factor-κB ligand (RANKL), osteoprotegerin (OPG), and IL-6 and TNF-α expression
	*In vivo*/*in vitro*	Curcumin	Suppression of osteoclast differentiation, MMP-9 expression, myeloperoxidase function, and reduction of alveolar bone loss
	*In vivo*	Curcumin	Reduction of alveolar bone loss and IL-1β level, and elevation of osteoblast number
	*In vitro*	Curcumin gel	Decrease of plaque formation, pocket depth, and bleeding indices
	Human	Curcumin gel	Decrease of the count of *Capnocytophaga*, *F. nucleatum*, *P. intermedia*, and *P. gingivalis* bacteria and reduction of bleeding index
	Human	Curcumin gel	Decrease of TNF-α, IL-1β and copper levels and plaque, bleeding, gingival, clinical attachment, and pocket depth indices, and elevation of magnesium and zinc levels
	*In vivo*	Curcumin gel	Reduction of inflammatory infiltration and IL-1β and RANKL levels, and osteoclast number
	Human	Curcumin gel	Decrease of gingival inflammation
	Human	Curcumin gel	Reduction of plaque, gingival, and pocket depth indices
			
Table 2. Continue			
	*In vitro*/human	Curcumin gel	Decrease of the activity of *T. denticola*, *T. forsythia*, and *P. gingivalis* bacteria and gingival, plaque, and pocket depth indices
	*In vitro*/human	Curcumin gel	Reduction of bleeding, pocket depth, clinical attachment, and plaque indices and the activity of *A. actinomycetemcomitans*, *P. intermedia*, and *P. gingivalis*
	*In vivo*	Curcumin gel	Reduction of pocket depth and gingival indices
	Human	Curcumin gel	Reduction of plaque, pocket depth, and clinical attachment indices
	*In vivo *	Triketonic phenylamino carbonyl curcumin	Reduction of alveolar bone loss and IL-1β level
	*In vivo*	Chemically modified curcumin	Decrease of inflammatory cell infiltrate, bone resorption, and osteoclast number
	*In vivo*	Chemically modified curcumin	Decrease of gingival and pocket depth indices and IL-1β, MMP-2, and MMP-9 levels, and alveolar bone loss
	*In vivo*/*in vitro*	Chemically modified curcumin	Inhibition of TNF-α and IL-1β release and reduction of MMP-9 release and alveolar bone loss
	*In vivo*	Curcumin solution	Diminution of osteoclastic function and inflammatory infiltration
	*In vivo*	Nanocurcumin	Reduction of the number of fibroblastic cells, bone resorption, osteoclast level, inflammatory infiltration, and NF-kB and p38 MAPK triggering
	Human	Nanocurcumin	Decrease of gingival and bleeding indices
	Human	Curcumin-loaded polyglycolic and poly-lactic acids (PGLA/PLA) nanoparticles	Reduction of IL-6 level
	*In vitro*	Quantum curcumin	Suppression of growth and biofilm formation of *P. gingivalis*, *S. mutans*, and *A. viscosus*

**Figure 2:**
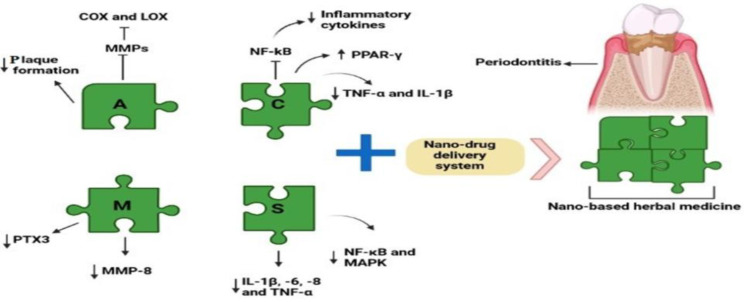
Nano-based herbal medicine using Aloe vera, curcumin, Melaleuca alternifolia, and Scutellaria baicalensis Georgi can significantly solve the pharmacological problems of herbal therapy and can ameliorate periodontitis through different mechanisms. COX, cyclooxygenase; LOX, lipoxygenase; NF-kB, Nuclear factor-kappa B; PPAR-γ, Peroxisome proliferator-activated receptor-γ; MMPs, Matrix metalloproteinases; MMP-8, Matrix metalloproteinase-8; TNF-α, Tumour necrosis factor-α; PTX3, Pentraxin 3; IL-1β, Interleukin 1β; IL-6, Interleukin 6; IL-8, Interleukin 8; The letters of A, C, M and B are the abbreviations of following plant names Respectively: Aloe vera, curcumin, Melaleuca alternifolia and Scutellaria baicalensis

Furthermore, systematic administration of curcumin (30 and 100 mg/kg) can result in the suppression of gene expression of PGE2, IL-6, and TNF-α and significant and dose-dependent inhibition of NF-kB activation in periodontitis *in vivo* (Guimarães et al., 2011 ▶). A research also proposed a crosslinked gelatin film, which is a biodegradable, mucoadhesive, and nontoxic material acquired by hydrolysis of animal connective tissues, bones, and skin, for loading curcumin to enhance periodontitis treatment (Chauhan et al., 2018 ▶; Perioli et al., 2004 ▶). This work implicated that this optimized film entraps curcumin without chemical and physical interactions. Plus, this formulation has suitable resistance and strength to forces and possesses enough flexibility to prevent an uncomfortable feel following its insertion into the periodontal pockets (Chauhan et al., 2018 ▶). Interestingly, it has been shown that curcumin gel injection (10 mg) into the periodontal pocket can increase magnesium and zinc levels in individuals with chronic periodontitis (Mohammad, 2020 ▶). These elements are crucial for the normal metabolism of lipids, carbohydrates, and proteins and act as antioxidant factors (Yamaguchi and Weitzmann, 2011 ▶). Curcumin can also exert its anti-inflammatory effect through the upregulation of peroxisome proliferator-activated receptor-*γ* (PPAR-*γ*) activation (Figure 2) (Jacob et al., 2007 ▶). PPAR-γ may curb bone resorption in periodontitis through the suppression of osteoclastogenesis induced by RANKL (Hassumi et al., 2009 ▶). Recently, the anti-inflammatory impacts of curcumin on the disease have been demonstrated by Justo et al. They revealed these effects through curcumin administration (once a day for 15 days, orally) in an animal model of apical periodontitis by reducing the levels of pro-inflammatory agents including IL-1β, TNF-α, and IL-6 (Justo et al., 2022 ▶). Moreover, a clinical study pointed out the mild benefits of subgingival use of curcumin gel in the decrement of gingival inflammation in chronic periodontitis patients (Kaur et al., 2019 ▶). Another clinical investigation highlighted the potential role of local curcumin gel in the reduction of sulcular bleeding, pocket depth, and plaque indices in patients with mild chronic periodontitis (Dave et al., 2018 ▶). However, one of the problems of curcumin is its low aqueous solubility and poor bioavailability. This problem can be solved through the preparation of curcumin-loaded NPs (Bhawana et al., 2011 ▶). In this line, Zambrano et al. investigated the effects of local utilization of curcumin-loaded NPs (0.05 mg/ml curcumin) in an animal model of periodontal disease induced by injecting LPS solution into the gingival tissue. They demonstrated that these NPs suppress inflammatory bone resorption and attenuate osteoclast levels and NF-kB (p65) and p38 MAPK function (Zambrano et al., 2018 ▶). In addition, an *in vitro* study addressed the possibility of the effectiveness of curcumin quantum dots (mean particle size 3.5 nm) on the suppression of growth and biofilm formation of periodontitis-related pathogens, such as *P. gingivalis*, *S. mutans*, and *Actinomyces viscosus* (Singh et al., 2018 ▶). Quantum dots are one of the nano-carriers for herbal products by coupling, dispersing, dissolving, and adsorption, etc. and can potentiate the bioavailability of drugs (Zhao et al., 2016 ▶). These nano-carriers enhance the penetration and interplay with the biofilm matrix and absorption by the bacterial cells (Singh et al., 2018 ▶). A double-blind randomized clinical trial also showed that the oral administration of nano-curcumin capsules (80 mg daily for 4 weeks) has favorable effects on gingival bleeding and the reduction of inflammation in subjects with mild periodontitis and gingivitis (Malekzadeh et al., 2021 ▶). In these capsules, spherical hydrophobic nanomicelles (∼10 nm size) encompassed all curcumin and could subsequently elevate the water solubility of curcumin (Malekzadeh et al., 2021 ▶). Taken together, nano-based formulations of curcumin, such as curcumin-loaded NPs and curcumin quantum dots, can elevate the effectiveness of this polyphenol against periodontitis by some mechanisms, like inhibiting bone resorption, inflammatory events, growth, and biofilm formation of disease-associated pathogens. 

***Melaleuca alternifolia*** **and its nanoformulations: Their effects on periodontitis**

**Figure 3:**
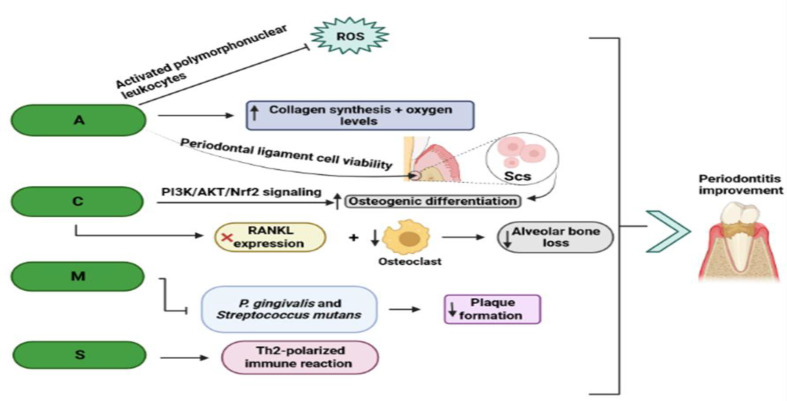
Aloe vera, curcumin, Melaleuca alternifolia, and Scutellaria baicalensis Georgi improve periodontitis mainly through the immune system regulation and reconstruction or viability of tissues and cells involved in the disease. The letters of A, C, M and B are the abbreviations of following plant names Respectively: Aloe vera, curcumin, Melaleuca alternifolia and Scutellaria baicalensis Georgi

Tea tree, another name for *Melaleuca alternifolia* (MEL), is an Australian plant with three main active components consisting of terpinen-4-ol, γ-terpinen, and 1,8-cineole and is used in herbal medicine due to its anti-bacterial and antifungal characteristics (Iiyama and Cardoso, 2021 ▶; Terzi et al., 2007 ▶). One of the products of this plant is Tea tree oil (TTO), which is derived through a steam distillation from this plant. TTO has antioxidant and broad-spectrum antimicrobial activity, especially against infections of the skin and mucosa. TTO can be utilized in the treatment of acne vulgaris and seborrheic dermatitis and in the improvement of the process of wound healing (Pazyar et al., 2013 ▶). Also, documents implicated a special ability of this plant in the treatment of oral pathogens and diseases (Francisconi et al., 2020 ▶; Hammer et al., 2003 ▶; Yadav et al., 2017 ▶). In this respect, an *in vitro *investigation approved the role of TTO in the inhibition of adherence of *A. actinomycetemcomitans* and *P. gingivalis* biofilms to enamel surfaces of premolar teeth (Soulissa et al., 2020 ▶). Regarding its anti-bacterial effects, some reports manifested that TTO may attenuate plaque formation through the suppression of *P. gingivalis *and* S. mutans *adhesion (Figure 3) (Raut and Sethi, 2016 ▶). Moreover, Raut and Sethi implicated the positive action of TTO gel administration (5 ml TTO was combined with methylcellulose gel) locally on subjects with chronic periodontitis by diminishing clinical attachment and pocket probing depth indices (Raut and Sethi, 2016 ▶). Similarly, a randomized controlled clinical research indicated that local application of TTO gel (5 ml TTO was mixed into methylcellulose gel) reduced pocket probing depth index and PTX3 level in subjects with periodontitis. PTX3 has a direct relationship with the levels of TNF and IL-1 and the number of bacterial products (Elgendy et al., 2013 ▶). Another clinical study by Taalab and colleagues revealed the striking role of local use of TTO 5% gel in the enhancement of periodontitis-related clinical parameters, including pocket depth, gingival, bleeding, and clinical attachment indices, and reduction of levels of MMP-8 (Figure 2), the main cause of the destruction of type I, II and III collagen, which in turn results in the reduction of disease severity (Taalab et al., 2021 ▶). MMP-8 is considered the main enzyme in the salivary fluid and gingival tissue that plays a substantial role in the destruction of the periodontal tissues (Taalab et al., 2021 ▶). In spite of various therapeutic effects of MEL recorded in papers, this Australian plant has some pharmacological restrictions, like high oil oxidation and volatility and low solubility (Battisti et al., 2021 ▶). To overcome these restrictions and improve the curative ability of MEL, Souza et al. in an *in situ* study, evaluated the antimicrobial influences NPs of 0.3% TTO on dental biofilm (de Souza et al., 2017 ▶). In this project, the results of analyzing the biofilm structure approved the better effectiveness of TTO NPs than TTO on biofilm formation (de Souza et al., 2017 ▶). The anti-bacterial activity of MEL NPs against *P. aeruginosa *and *Candida* species has also been reported in some articles (Comin et al., 2016 ▶; de Souza et al., 2017 ▶). Plus, MEL NPs can exert an anti-inflammatory effect in mouthwash. In this regard, a clinical study addressed this result using the synthesis of nano-based lipid carriers by 7.5% weight/volume (w/v) of MEL through high-pressure homogenization (Casarin et al., 2019 ▶). So, harnessing nano-based products of this plant, for example, NPs and nano-based lipid carriers may be a good therapeutic candidate for periodontitis by exerting anti-inflammatory and anti-bacterial effects. 

***Scutellaria baicalensis Georgi*** **and its nanoformulations: Their effects on periodontitis**

**Figure 4:**
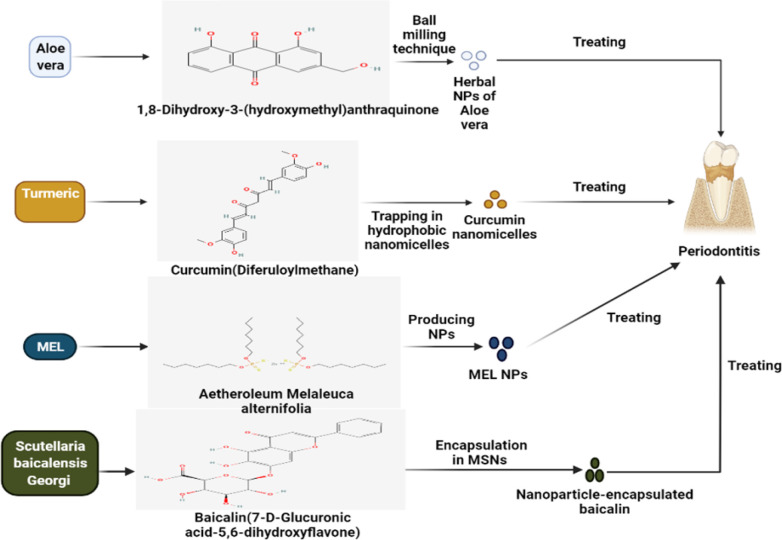
Different methods of preparation of nano-based herbal formulations from* Aloe vera*, curcumin, *Melaleuca alternifolia*, and *Scutellaria baicalensis Georgi *in order to treat periodontitis

*Scutellaria baicalensis Georgi *(Lamiaceae) is a plant of the Lamiaceae family, which is used in herbal medicine and mainly found in Asian countries (Wang et al., 2018 ▶; Zhao et al., 2019 ▶). *Lamiaceae *possesses many substances, and its main active substances include baicalein, baicalin, wogonin, wogonoside, and oroxylin A (Liao et al., 2021 ▶). This herb has antiviral, anti-oxidative, anti-inflammatory, immunoregulatory, neuroprotective, anti-microbial, hepatoprotective, and antineoplastic effects (Huang et al., 2013 ▶; Wang et al., 2018 ▶; Ye et al., 2009 ▶). Also, baicalin, as a flavonoid compound in this herb, has anti-periodontitis effects by modulating the expression of some pro-inflammatory factors in the process of periodontitis (Ming et al., 2018 ▶). Baicalein reflects its ani-inflammatory and osteogenic activities by diminishing the expression of IL-1β, MMP-1, MMP-2, TNF-α, and MCP-1 and upregulating osteogenic landmarks, like collagen-I, runt-related transcription factor 2 (RUNX2), and osterix, in periodontal ligament cells *in vitro *(Ren et al., 2021 ▶). Furthermore, an *in vivo* study assessed the impacts of intragastric exploitation of baicalin in a rat model of periodontitis induced by ligating the maxillary second molars and inoculating with *P. gingivalis* (Sun et al., 2016 ▶). This research concluded that baicalin (100 and 200 mg/kg/day) remarkably decreases alveolar bone loss, myeloperoxidase expression, the levels of IL-1β, TNF-α, high mobility group box 1 protein (HMGB1), and infiltration of inflammatory agents in gingival tissue (Sun et al., 2016 ▶). Another *in vivo* project by Kim and co-workers demonstrated that oral administration of *Lamiaceae* extract (100 mg/kg) reduces alveolar bone resorption, mRNA expression of IL-6 and IL-8, and suppresses cementum mineralization in periodontitis rats induced (Kim et al., 2018 ▶). Moreover, the aqueous extract of *Lamiaceae* (50 mg/kg/day, orally) can be a good therapeutic option in mouse models with periodontitis through the stimulation of Th2-polarized immune reaction, diminution of alveolar bone loss, and accumulation of collagen fiber (Huang et al., 2013 ▶). Despite all of the benefits of *Lamiaceae*, it has some pharmacological problems, such as low solubility, poor bioavailability, and short half-life, that impairs their biomedical applications (Wang et al., 2015 ▶; Xing et al., 2005 ▶). One of the suitable choices for solving this issue can be the encapsulation of baicalin and baicalein in synthesized mesoporous silica nanoparticles (MSNs) (Figure 4) ( Li et al., 2017 ▶). MSNs possess a spherical shape with ordered pore structures (the mean diameter 367±94 nm) and are among the important drug carriers because of their stability and high biocompatibility, and low cytotoxicity (Li et al., 2017 ▶; Ma et al., 2014 ▶). By harnessing this process, Li et al. expressed that nano-encapsulated baicalein can be a potential candidate against periodontitis by reducing the expression of pro-inflammatory cytokines, e.g. IL-6 and IL-8 (Li et al., 2017 ▶). It looks like the co-use of *Lamiaceae *and nano-based materials, like MSN, may have positive impacts on this oral disorder; however, this hypothesis needs more evidence.

**Some other herbal and nano-based herbal products effective in** **periodontitis** **treatment**

Some other herbal and nano-based herbal formulations have also reflected their capacity to treat periodontitis, like *Camellia sinensis*, resveratrol*, *and quercetin (Elagbar et al., 2020 ▶; Mallikarjun et al., 2016 ▶; Maurya et al., 1997 ▶; Mazur et al., 2021 ▶; Sezer et al., 2013 ▶; Warad et al., 2013 ▶). *Camellia sinensis* leaves, another name for green tea, can have positive effects on periodontitis treatment by reducing bleeding, gingival, plaque, clinical attachment, pocket depth indices, inflammation, alveolar bone loss, and osteoclastic function and increasing total antioxidant capacity (TAOC) and glutathione-S-transferase (GST) (de Almeida et al., 2019 ▶; Hrishi et al., 2016 ▶; Taleghani et al., 2018 ▶). Harnessing NPs of EGCG (one of the main active components of *green tea*) may also lead to the decrement of ROS levels and downregulation of expression of pro-inflammatory cytokines through the regulation of macrophages from the phenotypes of M1 to M2. Plus, this nano-based compound is capable of reducing osteoclast activity and suppressing alveolar bone loss in animal cases of chronic periodontitis (Tian et al., 2022 ▶). Resveratrol, a phenolic compound present in mulberries, peanuts, and red wines, is another plant compound effective in periodontitis therapy (Jang et al., 1997 ▶; Zhen et al., 2015 ▶). In this line, an experimental work indicated the inhibition of TNF-α, IL-8, TLR4, IL-6, and IL-1β levels in the gingival tissue of periodontitis mice receiving resveratrol (20 mg/kg, gavage administration) (Zhen et al., 2015 ▶). Shi and colleagues observed similar findings after using a liposomal system loaded with resveratrol. They reported that the utilization of this system reduced ROS levels and IL-1β, TNF-α, and IL-6 production owing to the inhibition of inflammasomes and the NF-κB signaling pathway (Shi et al., 2021 ▶). Liposomes are described as spherical vesicles comprising one or more lipid bilayer membranes that enhance the bioavailability, solubility, and function of active substances. They also curb biological and physicochemical degradation of delivered drugs, decrease toxicity and side effects of delivered drugs, and monitor their content release (Delma et al., 2021 ▶). In other attempts, the anti-periodontitis effects of quercetin, the most frequent flavonoid existent in different fruits and vegetables, have been securitized (Geoghegan et al., 2010 ▶; He et al., 2020 ▶). For instance, several *in vivo* and *in vitro* studies approved the striking role of this flavonoid in the diminution of oxidative stress level, alveolar bone absorption, L-1β, L-17, and TNF-α secretion, and suppression of growth of *P. gingivalis* and *A. actinomycetemcomitans* (Geoghegan et al., 2010 ▶; Napimoga et al., 2013 ▶; Taskan and Gevrek, 2020 ▶; Wei et al., 2021 ▶). Some researchers also utilized nano-based drug delivery methods, like nanoemulgel and ceria nanocomposite, for improving the pharmacological and therapeutic functions of quercetin against the disease (Aithal et al., 2018 ▶; Wang et al., 2021 ▶). In this line, the findings of Wang et al. revealed suppression of M1 macrophage polarization and enhancement of M2 macrophage polarization as a result of using a ceria nanocomposite loaded with quercetin in rats with periodontitis (Wang et al., 2021 ▶). Nanoceria is an appropriate nanomaterial with anti-oxidative and anti-inflammatory impacts in light of reversible transitions between ions of Ce^3+^ and Ce ^4+^ in the time of redox reaction and decrease of pro-inflammatory release (Luo et al., 2020 ▶; Wang et al., 2021 ▶). Moreover, Aithal and colleagues showed that quercetin nanoemulgel developed by cinnamon oil, Carbitol® and poloxamer 407, and tween 80 have suitable physical properties, syringeability, stability, and sol-gel transition, and thus, this nanoformulation can be utilized in periodontitis profitably. On the whole, different nano-based herbal formulations have addressed their ability to overcome herbal therapy limitations and ameliorate periodontitis through various mechanisms (Tables 3 and 4). However, more investigations are thought to be needed to approve these findings.

**Table 3:** Some other herbal and nano-based herbal products effective in periodontitis treatment

**Type of plant**	**Effect/mechanism**	**In vivo/in vitro/ human**	**Reference**
*Nano-emulsion of mangosteen rind extract*	Decrease of TNF-a and RANKL expression and increase of IL-10 expression	In vivo	
*Propolis extract*	Reducing the subgingival plaque formation and microbiota from periodontal pockets	In vivo	
*Crocus sativus L.*	Anti-inflammatory effects, strong antioxidant properties and the ability to accumulate oxygen free radicals	In vivo	
*Eucalyptus globulus leaf, Azadirachta indica leaf*	Antimicrobial activity against porphyromonas gingivalis	In vitro	Müller-Heupt et al.

**Table 4:** Some of clinical trials for application of herbal medicine in dentistry

**Ref.**	**Result**	**Method**	**Number of** **participants**	**Product**
(Armidin and Yanti,2019 ▶)	In comparison, green tea herbal mouth rinse showed higher efficacy in reducing S. mutans count than black tea mouth rinse	Comparison of *S. mutans *bacterial count found in saliva before and after *C. sinensis *mouth rinses administration	60	*Green and black tea* *mouth rinse*
(Salama and Alsughier,2019 ▶(	C. sinensis extract mouth rinse caused a significant decrease in S. mutans bacterial load in saliva	Quantitative microbiological laboratory cultivation assay. Comparison of S. mutans bacterial count found in saliva before and after mouth rinse administration in pre-school children	40	*Herbal mouthwash*
(Patri and Sahu,2017 ▶(	CHX as a control group exerted the strongest efficacy against cariogenic microorganisms followed by M. alternifolia	Evaluating the anti-microbial efficacy after caries excavation and topical application of herbal medicaments on dentinal specimens by total viable count analysis	40	*Tea tree oil/Aloe vera* *gel*
(Bhat et al.,2017 ▶(	Using herbal mouthwash significantly reduced S. mutans count but not as well as CHX	RCT/Comparison of S. mutans bacterial load detected in saliva before and after herbal mouth rinse and CHX administration by colony-forming units count	20	*mango leaf mouthwash*
(Yadav et al.,2017 ▶(	C. Arabica showed the same efficacy as CHX in decreasing S. mutans salivary load	Parallel RCT/Comparison of S. mutans bacterial load detected in saliva before and after herbal mouth rinse and CHX administration by colony-forming units count	45	*Herbal extract*
(Saxena et al.,2017 ▶)	All groups showed significant antimicrobial efficacy but the highest results were obtained by using the combination of all herbal extracts	Linear randomized cross over study/ Comparison of S. mutans bacterial load detected in saliva before and after herbal mouth rinses individually and in combination by colony-forming units count	40	*Herbal aqueous* *extracts and Triphala*
(Abdelmegid et al.,2015 ▶(	Administration of C. sinensis mouthwash significantly reduced salivary S. mutans count	Cross-sectional study/Comparison of S. mutans bacterial load detected in saliva before and after herbal mouth rinses in school children by colony-forming units count	30	*Herbal mouthwash*
(Casarin et al.,2019 ▶(	The herbal mouth rinse showed the same anti-inflammatory efficacy compared to CHX without affecting taste sensation	Double-blinded crossover RCT/gingival crevicular fluid volume and the Quigley & Hein plaque index comparison before and after herbal mouthwash administration	60	*M. alternifolia* *nanoparticle*
(Pradeep et al.,2016 ▶(	A significant improvement of periodontal indices was recorded in the herbal mouth rinse group	Double-blinded RCT/Oral hygiene index-simplified, PI, and GI comparison before and after using an herbal mouth rinse	90	*Triphala mouthwash*
(Purohit et al.,2017 ▶(	Pulpotomy treatment using turmeric powder in primary teeth resulted in proper clinical and radiographic success	Clinical and Radiological Evaluation was conducted after using turmeric powder for primary teeth pulpotomy medicament	15	*Curcuma longa*
(Songsiripradubboon et al., 2016 ▶)	Compared to calcium hydroxide, using acemannan as a direct pulp capping agent resulted in better histological responses and biocompatibility	Clinical, radiographic, and histologic analyses after direct pulp capping treatment with the herbal agent in primary teeth	42	*Aloe vera*
(Nimma et al.,2017 ▶)	Aloe vera extract can be used as an adjunct therapy agent for socket healing improvement after dental extraction	Cross-sectional randomized interventional method/standardized index by Landry, Turnbull, and Howley assessment after third molar surgery in patients treated with foam gel soaked in Aloe vera extract	40	*Aloe vera*

## Discussion

Periodontitis, as an inflammatory condition related to tooth-supporting tissues, affects a large number of subjects over the world, and unfortunately, common therapies have not reflected enough effectiveness with minimum side effects. Nowadays, popular herbal products, particularly *Aloe vera*, Curcumin, *Melaleuca alternifolia*, and *Scutellaria baicalensis Georgi*, have highlighted their abilities in the treatment of periodontitis, by improving clinical parameters, like bleeding, pocket depth, plaque, and clinical attachment indices. Also, from a mechanistic point of view, these popular herbal products target periodontitis through several mechanisms, such as suppression of COX and LOX, NF-kB signaling pathway, reduction of levels of MMP-1, MMP-2, MMP-8, MCP-1, and the expression of some inflammatory agents (e.g., IL-1β, IL-6, IL-8, and TNF-α) which all are involved in periodontitis pathogenesis directly or indirectly. However, these herbal remedies may have some pharmacological problems, such as low aqueous solubility, short half-life, and low bioavailability. On the other hand, it has been shown that the co-use of herbal medicine and nano-based formulations (like NPs, MSN, nano-based lipid carriers, and quantum dots), not only can overcome the limitation of herbal therapy but also are capable of improving periodontitis. Thus, nano-based herbal products can create a good chance to treat the disease efficiently. However, more experimental and clinical investigations are required to validate these findings. 

## Conflicts of interest

The authors have declared that there is no conflict of interest.

## References

[B1] Aljuanid MA, Qaid HR, Lashari DM, Ridwan RD, Budi HS, Alkadasi BA, Ramadhani Y, Rahmasari R (2022). Nano-emulsion of mangosteen rind extract in a mucoadhesive patch for periodontitis regenerative treatment: An in vivo study. J Taibah Univ Medical Sci.

[B2] Abdelmegid F, Al-Agamy M, Alwohaibi A, Ka'abi H, Salama F (2015). Effect of honey and green tea solutions on Streptococcus mutans. J Clin Pediatr Dent.

[B3] Abdelmonem HM, Khashaba OH, Al-Daker MA, Moustafa MD (2014). Effects of aloe vera gel as an adjunctive therapy in the treatment of chronic periodontitis: A clinical and microbiologic study. Mansoura J Dent.

[B4] Adam AM, Achmad MH, Fahruddin AM (2018). Efficacy of mouthwash from aloe vera juice after scaling treatment on patient with gingivitis: A clinical study. Pesqui Bras Odontopediatria Clin Integr.

[B5] Aggarwal B, Prasad S, Reuter S, Kannappan R, R Yadav V, Park B, Hye Kim J, Gupta S, Phromnoi K, Sundaram C, Prasad S (2011). Identification of novel anti-inflammatory agents from Ayurvedic medicine for prevention of chronic diseases:“reverse pharmacology” and “bedside to bench” approach. Curr Drug Targets.

[B6] Aithal GC, Nayak UY, Mehta C, Narayan R, Gopalkrishna P, Pandiyan S, Garg S (2018). Localized in situ nanoemulgel drug delivery system of quercetin for periodontitis: development and computational simulations. Molecules.

[B8] Akpinar A, Calisir M, Cansın Karakan N, Lektemur Alpan A, Goze F, Poyraz O (2017). Effects of curcumin on alveolar bone loss in experimental periodontitis in rats: A morphometric and histopathologic study. Int J Vitam Nutr Res.

[B9] Al‐Maweri SA, Alhajj MN, Deshisha EA, Alshafei AK, Ahmed AI, Almudayfi NO, Alshammari SA, Alsharif A, Kassim S (2022). Curcumin mouthwashes versus chlorhexidine in controlling plaque and gingivitis: A systematic review and meta‐analysis. Int J Dent Hyg.

[B10] Álvarez AL, Espinar FO, Méndez JB (2011). The application of microencapsulation techniques in the treatment of endodontic and periodontal diseases. Pharmaceutics.

[B11] Aoki‐Nonaka Y, Nakajima T, Miyauchi S, Miyazawa H, Yamada H, Domon H, Tabeta K, Yamazaki K (2014). Natural killer T cells mediate alveolar bone resorption and a systemic inflammatory response in response to oral infection of mice with P orphyromonas gingivalis. J Periodontal Res.

[B12] ArefNezhad R, Motedayyen H, Roghani-Shahraki H (2022). Do cytokines associate periodontitis with metabolic disorders? An overview of current documents. Endocr Metab Immune Disord Drug Targets.

[B13] Armidin RP, Yanti GN (2019). Effectiveness of rinsing black tea compared to green tea in decreasing streptococcus mutans. Open Access Maced J Med Sci.

[B14] Ashouri Moghaddam A, Radafshar G, Jahandideh Y, Kakaei N (2017). Clinical evaluation of effects of local application of Aloe vera gel as an adjunct to scaling and root planning in patients with chronic periodontitis. J Dent (Shiraz).

[B15] Bapat RA, Chaubal TV, Joshi CP, Bapat PR, Choudhury H, Pandey M, Gorain B, Kesharwani P (2018). An overview of application of silver nanoparticles for biomaterials in dentistry. Mater Sci Eng C Mater Biol Appl.

[B16] Barkat MA, Harshita D, Beg S, Ahmad FJ, Beg S, Barkat M (2020). Nanotechnology-Based Phytotherapeutics: Current Status and Challenges. Nanophytomedicine.

[B17] Bascones Martínez A, Muñoz Corcuera M, Noronha S, Mota P, Bascones Ilundain C, Campo Trapero J (2009). Host defence mechanisms against bacterial aggression in periodontal disease: Basic mechanisms. Med Oral Patol Oral Cir Bucal.

[B18] Battisti MA, Caon T, de Campos AM (2021). A short review on the antimicrobial micro-and nanoparticles loaded with Melaleuca alternifolia essential oil. J Drug Deliv Sci Technol.

[B19] Bedi MK, Shenefelt PD (2002). Herbal therapy in dermatology. Arch dermatol.

[B20] Bhat G, Kudva P, Dodwad V (2011). Aloe vera: Nature's soothing healer to periodontal disease. J Indian Soc Periodontol.

[B21] Bhat SS, Hegde KS, Mathew C, Bhat SV, Shyamjith M (2017). Comparative evaluation of Mangifera indica leaf mouthwash with chlorhexidine on plaque accumulation, gingival inflammation, and salivary streptococcal growth. Indian J Dent Res.

[B22] Bhatia M, Urolagin SS, Pentyala KB, Urolagin SB, KB M, Bhoi S (2014). Novel therapeutic approach for the treatment of periodontitis by curcumin. J Clin Diagnostic Res.

[B23] Basniwal RK, Buttar HS, Jain VK, Jain N (2011). Curcumin nanoparticles: preparation, characterization, and antimicrobial study. J Agric Food Chem.

[B24] Buwalda SJ, Vermonden T, Hennink WE (2017). Hydrogels for therapeutic delivery: current developments and future directions. Biomacromolecules.

[B25] Cairo F, Nieri M, Gori AM, Tonelli P, Branchi R, Castellani S, Abbate R, Pini-Prato GP (2010). Markers of systemic inflammation in periodontal patients: chronic versus aggressive periodontitis An explorative cross-sectional study. Eur J Oral Implantol.

[B26] Casarin M, Pazinatto J, Santos RCV, Zanatta FB (2018). Melaleuca alternifolia and its application against dental plaque and periodontal diseases: A systematic review. Phytother Res.

[B27] Casarin M, Pazinatto J, Oliveira LM, Souza ME, Santos RC, Zanatta FB (2019). Anti-biofilm and anti-inflammatory effect of a herbal nanoparticle mouthwash: a randomized crossover trial. Braz Oral Res.

[B28] Casarin M, Pazinatto J, Oliveira LM, Souza MED, Santos RCV, Zanatta FB (2019). Anti-biofilm and anti-inflammatory effect of a herbal nanoparticle mouthwash: a randomized crossover trial. Braz Oral Res.

[B29] Catanzaro M, Corsini E, Rosini M, Racchi M, Lanni C (2018). Immunomodulators inspired by nature: a review on curcumin and echinacea. Molecules.

[B30] Choudhary M, Kochhar A, Sangha J (2014). Hypoglycemic and hypolipidemic effect of Aloe vera L in non-insulin dependent diabetics. J Food Sci Technol.

[B31] Chaudhari PM, Randive SR (2020). Incorporated herbal drugs in novel drug delivery system. Asian J Pharm Pharmacol.

[B32] Chauhan S, Bansal M, Khan G, Yadav SK, Singh AK, Prakash P, Mishra B (2018). Development, optimization and evaluation of curcumin loaded biodegradable crosslinked gelatin film for the effective treatment of periodontitis. Drug Dev Ind Pharm.

[B33] Choi S, Chung M-H (2003). A review on the relationship between aloe vera components and their biologic effects. Semin Integr Med.

[B34] Comin VM, Lopes LQ, Quatrin PM, de Souza ME, Bonez PC, Pintos FG, Raffin RP, Vaucher RD, Martinez DS, Santos RC (2016). Influence of Melaleuca alternifolia oil nanoparticles on aspects of Pseudomonas aeruginosa biofilm. Microb Pathog.

[B35] Corrêa MG, Pires PR, Ribeiro FV, Pimentel SZ, Casarin RC, Cirano FR, Tenenbaum HT, Casati MZ (2017). Systemic treatment with resveratrol and/or curcumin reduces the progression of experimental periodontitis in rats. J Periodontal Res.

[B36] Craig R (2008). Interactions between chronic renal disease and periodontal disease. Oral Dis.

[B37] Curylofo-Zotti FA, Elburki MS, Oliveira PA, Cerri PS, Santos LA, Lee H-M, Johnson F, Golub LM, Junior CR, Guimarães-Stabili MR (2018). Differential effects of natural Curcumin and chemically modified curcumin on inflammation and bone resorption in model of experimental periodontitis. Arch oral biol.

[B38] Damiano S, Longobardi C, Andretta E, Prisco F, Piegari G, Squillacioti C, Montagnaro S, Pagnini F, Badino P, Florio S, Ciarcia R (2021). Antioxidative effects of curcumin on the hepatotoxicity induced by ochratoxin a in rats. Antioxidants.

[B39] Dave DH, Patel P, Shah M, Dadawala SM, Saraiya K, Sant AV (2018). Comparative evaluation of efficacy of oral curcumin gel as an adjunct to scaling and root planing in the treatment of chronic periodontitis. Adv Hum Biol.

[B40] Deepu S, Kumar K, Nayar BR (2018). Efficacy of Aloe vera gel as an adjunct to scaling and root planing in management of chronic localized moderate periodontitis: A randomized clinical trial. Int J Oral Care Res.

[B41] Delma KL, Lechanteur A, Evrard B, Semdé R, Piel G (2021). Sterilization methods of liposomes: Drawbacks of conventional methods and perspectives. Int J Pharm.

[B42] de Almeida JM, Marques BM, Novaes VCN, de Oliveira FLP, Matheus HR, Fiorin LG, Ervolino E (2019). Influence of adjuvant therapy with green tea extract in the treatment of experimental periodontitis. Arch Oral Biol.

[B43] Deng J, Golub LM, Lee HM, Lin MC, Bhatt HD, Hong HL, Johnson F, Scaduto J, Zimmerman T, Gu Y (2020). Chemically-modified curcumin 2 24: A novel systemic therapy for natural periodontitis in Dogs. J Exp Pharmacol.

[B44] de Souza ME, Clerici DJ, Verdi CM, Fleck G, Quatrin PM, Spat LE, Bonez PC, Dos Santos CF, Antoniazzi RP, Zanatta FB, Gundel A (2017). Antimicrobial activity of Melaleuca alternifolia nanoparticles in polymicrobial biofilm in situ. Microb pathog.

[B45] Elburki MS, Rossa C, Guimaraes MR, Goodenough M, Lee HM, Curylofo FA, Zhang Y, Johnson F, Golub LM (2014). A novel chemically modified curcumin reduces severity of experimental periodontal disease in rats: initial observations. Mediators Inflamm.

[B46] Elagbar ZA, Shakya AK, Barhoumi LM, Al‐Jaber HI (2020). Phytochemical diversity and pharmacological properties of Rhus coriaria. Chem Biodivers.

[B47] Elgendy EA, Ali SAM, Zineldeen DH (2013). Effect of local application of tea tree (Melaleuca alternifolia) oil gel on long pentraxin level used as an adjunctive treatment of chronic periodontitis: A randomized controlled clinical study. J Indian Soc Periodontol.

[B48] Fakhri E, Eslami H, Maroufi P, Pakdel F, Taghizadeh S, Ganbarov K, ousefi M, Tanomand A, Yousefi B, Mahmoudi S, Kafil HS (2020). Chitosan biomaterials application in dentistry. Int J Biol Macromol.

[B49] Forouzanfar F, Forouzanfar A, Sathyapalan T, Orafai HM, Sahebkar A (2020). Curcumin for the management of periodontal diseases: A review. Curr Pharm Des.

[B50] Francisconi RS, Huacho PMM, Tonon CC, Bordini EAF, Correia MF, Sardi JdCO, Spolidorio DM (2020). Antibiofilm efficacy of tea tree oil and of its main component terpinen-4-ol against Candida albicans. Braz Oral Res.

[B51] Fulzele P, Baliga S, Thosar N, Pradhan D (2016). Evaluation of Aloevera gel as a storage medium in maintaining the viability of periodontal ligament cells - An in vitro study. J Clin Pediatr Dent.

[B52] Gao Y, Kuok KI, Jin Y, Wang R (2019). Biomedical applications of Aloe vera. Crit Rev Food Sci Nutr.

[B53] Gasner NS, Schure RS, Gasner NS, Schure RS (2022). Necrotizing Periodontal Disease. StatPearls.

[B54] Geoghegan F, Wong R, Rabie A (2010). Inhibitory effect of quercetin on periodontal pathogens in vitro. Phytother Res.

[B55] Gölz L, Memmert S, Rath-Deschner B, Jäger A, Appel T, Baumgarten G, Götz W, Frede S (2014). LPS from P. gingivalis and hypoxia increases oxidative stress in periodontal ligament fibroblasts and contributes to periodontitis. Med Inflamm.

[B56] Guimarães MR, Coimbra LS, de Aquino SG, Spolidorio LC, Kirkwood KL, Rossa Jr C (2011). Potent anti‐inflammatory effects of systemically administered curcumin modulate periodontal disease in vivo. J Periodontal Res.

[B57] Guimaraes MR, de Aquino SG, Coimbra LS, Spolidorio LC, Kirkwood KL, Rossa Jr C (2012). Curcumin modulates the immune response associated with LPS-induced periodontal disease in rats. Innate Immun.

[B58] Guimaraes-Stabili MR, de Aquino SG, de Almeida Curylofo F, Tasso CO, Rocha FRG, de Medeiros MC, de Pizzol JP, Cerri PS, Romito GA, Rossa C (2019). Systemic administration of curcumin or piperine enhances the periodontal repair: a preliminary study in rats. Clin Oral Investig.

[B59] Guo X, Mei N (2016). Aloe vera: A review of toxicity and adverse clinical effects. J Environ Sci Health.

[B60] Gürkan A, Emingil G, Nizam N, Doğanavşargil B, Sezak M, Kütükçüler N, Atilla G (2009). Therapeutic efficacy of vasoactive intestinal peptide in Escherichia coli lipopolysaccharide‐induced experimental periodontitis in rats. J Periodontol.

[B61] Hajishengallis G (2015). Periodontitis: from microbial immune subversion to systemic inflammation. Nat Rev Immunol.

[B62] Hajishengallis G (2014). Immunomicrobial pathogenesis of periodontitis: keystones, pathobionts, and host response. Trends Immunol.

[B63] Hammer K, Dry L, Johnson M, Michalak E, Carson C, Riley T (2003). Susceptibility of oral bacteria to Melaleuca alternifolia (tea tree) oil in vitro. Oral Microbiol Immunol.

[B64] Hassumi MY, Silva-Filho VJ, Campos-Júnior JC, Vieira SM, Cunha FQ, Alves PM, Alves JB, Kawai T, Gonçalves RB, Napimoga MH (2009). PPAR-γ agonist rosiglitazone prevents inflammatory periodontal bone loss by inhibiting osteoclastogenesis. Int Immunopharmacol.

[B65] He Z, Zhang X, Song Z, Li L, Chang H, Li S, Zhou W (2020). Quercetin inhibits virulence properties of Porphyromas gingivalis in periodontal disease. Sci Rep.

[B66] Heta S, Robo I (2018). The side effects of the most commonly used group of antibiotics in periodontal treatments. Med Sci.

[B67] Hewlings SJ, Kalman DS (2017). Curcumin: a review of its effects on human health. Foods.

[B68] Hirschfeld J (2020). Neutrophil subsets in periodontal health and disease: A mini review. Front immunol.

[B69] Hrishi TS, Kundapur PP, Naha A, Thomas BS, Kamath S, Bhat GS (2016). Effect of adjunctive use of green tea dentifrice in periodontitis patients–A randomized controlled pilot study. Int J Dent Hyg.

[B70] Hosadurga RR, Rao SN, Jose J, Rompicharla NC, Shakil M, Shashidhara R (2014). Evaluation of the efficacy of 2% curcumin gel in the treatment of experimental periodontitis. Pharmacognosy Res.

[B71] Huang S, Huang Q, Huang B, Lu F (2013). The effect of Scutellaria baicalensis Georgi on immune response in mouse model of experimental periodontitis. J Dent Sci.

[B72] Hudwekar AD, Beldar A, Murkute S, Lendhey SS, Thamke M (2019). Aloe vera on wound healing after periodontal flap surgery in chronic periodontitis patient: A randomized control trial. J Oral Res Rev.

[B73] Irani FC, Wassall RR, Preshaw PM (2015). Impact of periodontal status on oral health-related quality of life in patients with and without type 2 diabetes. J Dent.

[B74] Jacob A, Wu R, Zhou M, Wang P (2008). Mechanism of the anti-inflammatory effect of curcumin: PPAR-γ activation. PPAR Res.

[B75] Jain N, Jain GK, Javed S, Iqbal Z, Talegaonkar S, Ahmad FJ, Khar RK (2008). Recent approaches for the treatment of periodontitis. Drug Discov Today.

[B76] Jang M, Cai L, Udeani GO, Slowing KV, Thomas CF, Beecher CW, Fong HH, Farnsworth NR, Kinghorn AD, Mehta RG, Moon RC (1997). Cancer chemopreventive activity of resveratrol, a natural product derived from grapes. J Sci.

[B77] Justo MP, Cardoso CBM, Cantiga-Silva C, de Oliveira PHC, Sivieri-Araújo G, Azuma MM, Ervolino E, Cintra LT (2022). Curcumin reduces inflammation in rat apical periodontitis. Int Endod J.

[B78] Kagiya T (2016). MicroRNAs: Potential biomarkers and therapeutic targets for alveolar bone loss in periodontal disease. Int J Mol Sci.

[B79] Kaur A, Kapoor D, Soni N, Gill S (2016). Phytodentistry–a boon. Arch dent med res.

[B80] Karim B, Bhaskar DJ, Agali C, Gupta D, Gupta RK, Jain A, Kanwar A (2014). Effect of Aloe vera mouthwash on periodontal health: triple blind randomized control trial. Oral Health Dent Manag.

[B81] Khogta S, Patel J, Barve K, Londhe V (2020). Herbal nano-formulations for topical delivery. J Herb Med.

[B82] Kim MH, Lee H, Choi YY, Lee DH, Yang WM (2018). Scutellaria baicalensis ameliorates the destruction of periodontal ligament via inhibition of inflammatory cytokine expression. J Chin Med Assoc.

[B83] Kolaparthy LK, Sanivarapu S, Swarna C, Devulapalli NS (2014). Neutrophil extracellular traps: Their role in periodontal disease. J Indian Soc Periodontol.

[B84] Kurian IG, Dileep P, Ipshita S, Pradeep AR (2018). Comparative evaluation of subgingivally-delivered 1% metformin and Aloe vera gel in the treatment of intrabony defects in chronic periodontitis patients: A randomized, controlled clinical trial. J Investig Clin Dent.

[B85] Lal J (2012). Turmeric, curcumin and our life: a review. Bull Env Pharmacol Life Sci.

[B86] Langmead L, Makins RJ, Rampton DS (2004). Anti‐inflammatory effects of aloe vera gel in human colorectal mucosa in vitro. Aliment Pharmacol Ther.

[B87] Lawrence R, Tripathi P, Jeyakumar E (2009). Isolation, purification and evaluation of antibacterial agents from Aloe vera. Braz J Microbiol.

[B88] Lertpimonchai A, Rattanasiri S, Vallibhakara SAO, Attia J, Thakkinstian A (2017). The association between oral hygiene and periodontitis: a systematic review and meta-analysis. Int Dent J.

[B89] Liao H, Ye J, Gao L, Liu Y (2021). The main bioactive compounds of Scutellaria baicalensis Georg for alleviation of inflammatory cytokines: A comprehensive review. Biomed Pharmacother.

[B90] Luo L-J, Nguyen DD, Lai J-Y (2020). Dually functional hollow ceria nanoparticle platform for intraocular drug delivery: A push beyond the limits of static and dynamic ocular barriers toward glaucoma therapy. Biomater.

[B91] Iova GM, Calniceanu H, Popa A, Szuhanek CA, Marcu O, Ciavoi G, Scrobota I (2021). The antioxidant effect of curcumin and rutin on oxidative stress biomarkers in experimentally induced periodontitis in hyperglycemic Wistar rats. Molecules.

[B92] Liu C, Mo L, Niu Y, Li X, Zhou X, Xu X (2017). The role of reactive oxygen species and autophagy in periodontitis and their potential linkage. Front Physiol.

[B93] Li X, Luo W, Ng TW, Leung PC, Zhang C, Leung KC-F, Jin L (2017). Nanoparticle-encapsulated baicalein markedly modulates pro-inflammatory response in gingival epithelial cells. Nanoscale.

[B94] Li Y, Jiao J, Qi Y, Yu W, Yang S, Zhang J, Zhao J (2021). Curcumin: A review of experimental studies and mechanisms related to periodontitis treatment. J Periodontal Res.

[B95] Ilyas S (2020). Histology of Spleen after Induction Nanoherbal Rhodomyrtus tomentosa. Int J Psychophysiol.

[B96] Iiyama CM, Cardoso JC (2021). Micropropagation of Melaleuca alternifolia by shoot proliferation from apical segments. Trees.

[B97] Ma Z, Bai J, Wang Y, Jiang X (2014). Impact of shape and pore size of mesoporous silica nanoparticles on serum protein adsorption and RBCs hemolysis. ACS Appl Mater.

[B98] Majidzadeh H, Araj-Khodaei M, Ghaffari M, Torbati M, Dolatabadi JEN, Hamblin MR (2020). Nano-based delivery systems for berberine: A modern anti-cancer herbal medicine. Colloids Surf B.

[B99] Malekzadeh M, Kia SJ, Mashaei L, Moosavi MS (2021). Oral n ano‐curcumin on gingival inflammation in patients with gingivitis and mild periodontitis. Clin Exp Dent.

[B100] Mallikarjun S, Rao A, Rajesh G, Shenoy R, Pai M (2016). Antimicrobial efficacy of Tulsi leaf (Ocimum sanctum) extract on periodontal pathogens: An in vitro study. J Indian Soc Periodontol.

[B101] Mamillapalli V (2016). Nanoparticles for herbal extracts. Asian J Pharm.

[B102] Mariano FS, Sardi JDCO, Duque C, Höfling JF, Gonçalves RB (2010). The role of immune system in the development of periodontal disease: a brief review. Rev Odonto Cienc.

[B103] Maurya D, Mittal N, Sharma K, Nath G (1997). Role of triphala in the management of peridontal disease. Anc Sci Life.

[B104] Mau L-P, Cheng W-C, Chen J-K, Shieh Y-S, Cochran DL, Huang R-Y (2016). Curcumin ameliorates alveolar bone destruction of experimental periodontitis by modulating osteoclast differentiation, activation and function. J Funct Foods.

[B105] Mayadas TN, Tsokos GC, Tsuboi N (2009). Mechanisms of immune complex–mediated neutrophil recruitment and tissue injury. Circulation.

[B106] Maybodi FR, Vaziri F, Ghanbarnezhad S, Herandi V (2022). The effect of aqueous extract of Crocus sativus L (saffron) on periodontal indices of patients with generalized periodontitis. J Tradit. Complement Med.

[B107] Mazur M, Ndokaj A, Jedlinski M, Ardan R, Bietolini S, Ottolenghi L (2021). Impact of Green Tea (Camellia Sinensis) on periodontitis and caries Systematic review and meta-analysis. Jpn Dent Sci Rev.

[B108] Meyle J, Dommisch H, Groeger S, Giacaman RA, Costalonga M, Herzberg M (2017). The innate host response in caries and periodontitis. J Clin Periodont.

[B109] Ming J, Zhuoneng L, Guangxun Z (2018). Protective role of flavonoid baicalin from Scutellaria baicalensis in periodontal disease pathogenesis: a literature review. Complement Ther Med.

[B110] Mohammad CA (2020). Efficacy of curcumin gel on zinc, magnesium, copper, IL-1β, and TNF-α in chronic periodontitis patients. Biomed Res Int.

[B111] Mokhtar RH, Korany NS, Taha NS, Abbas M (2016). Oral or injectable aloe vera? Approaches for treating gingivitis associated with ligature induced periodontitis in wistar rats. Egypt Dent J.

[B112] Motterle A, Pu X, Wood H, Xiao Q, Gor S, Liang Ng F (2012). Functional analyses of coronary artery disease associated variation on chromosome 9p21 in vascular smooth muscle cells. Hum Mol Genet.

[B113] Müller-Heupt LK, Vierengel N, Groß J, Opatz T, Deschner J, von Loewenich FD (2022). Antimicrobial activity of Eucalyptus globulus, Azadirachta indica, Glycyrrhiza glabra, Rheum palmatum extracts and rhein against Porphyromonas gingivalis. Antibiotics.

[B114] Nagasri M, Madhulatha M, Musalaiah SVVS, Kumar PA, Krishna CHM, Kumar PM (2015). Efficacy of curcumin as an adjunct to scaling and root planning in chronic periodontitis patients: A clinical and microbiological study. J Pharm Bioallied Sci.

[B115] Napimoga MH, Clemente-Napimoga JT, Macedo CG, Freitas FF, Stipp RN, Pinho-Ribeiro FA, Casagrande R, Verri Jr WA (2013). Quercetin inhibits inflammatory bone resorption in a mouse periodontitis model. J Nat Prod.

[B116] Nasra MM, Khiri HM, Hazzah HA, Abdallah OY (2017). Formulation, in-vitro characterization and clinical evaluation of curcumin in-situ gel for treatment of periodontitis. Drug Deliv.

[B117] Nejatzadeh-Barandozi F (2013). Antibacterial activities and antioxidant capacity of Aloe vera. Org Med Chem Lett.

[B118] Nilsson H, Berglund JS, Renvert S (2018). Periodontitis, tooth loss and cognitive functions among older adults. Clin Oral Investig.

[B119] Nimma VL, Talla HV, Bairi JK, Gopaldas M, Bathula H, Vangdoth S (2017). Holistic healing through herbs: effectiveness of aloe vera on post extraction socket healing. J Clin Diagn Res.

[B120] Olsen I, Taubman MA, Singhrao SK (2016). Porphyromonas gingivalis suppresses adaptive immunity in periodontitis, atherosclerosis, and Alzheimer’s disease. J Oral Microbiol.

[B121] Passariello C, Lucchese A, Virga A, Pera F, Gigola P (2012). Isolation of Staphylococcus aureus and progression of periodontal lesions in aggressive periodontitis. Eur J Inflamm.

[B122] Patri G, Sahu A (2017). Role of herbal agents-tea tree oil and aloe vera as cavity disinfectant adjuncts in minimally invasive dentistry-an in vivo comparative study. J Clin Diagn Res.

[B123] Pazyar N, Yaghoobi R, Bagherani N, Kazerouni A (2013). A review of applications of tea tree oil in dermatology. Int J Dermatol.

[B124] Penmetsa GS, Vivek B, Bhupathi AP, Sudha Rani P (2019). Comparative evaluation of Triphala, Aloe vera, and chlorhexidine mouthwash on gingivitis: A randomized controlled clinical trial. Contemp Clin Dent.

[B125] Penmetsa GS, Subbareddy B, Mopidevi A, Arunbhupathi P, Baipalli V, Pitta S (2019). Comparing the effect of combination of 1% ornidazole and 0 25% chlorhexidine gluconate (Ornigreat™) gel and Aloe vera gel in the treatment of chronic periodontitis: A randomized, single-blind, split-mouth study. Contemp Clin Dent.

[B126] Pérez-Pacheco CG, Fernandes NAR, Primo FL, Tedesco AC, Bellile E, Retamal-Valdes B, Feres M, Guimarães-Stabili MR, Rossa C (2021). Local application of curcumin-loaded nanoparticles as an adjunct to scaling and root planing in periodontitis: Randomized, placebo-controlled, double-blind split-mouth clinical trial. Clin Oral Investig.

[B127] Perioli L, Ambrogi V, Rubini D, Giovagnoli S, Ricci M, Blasi P, Rossi C (2004). Novel mucoadhesive buccal formulation containing metronidazole for the treatment of periodontal disease. J Control Release.

[B128] Pimentel SP, Casati MZ, Ribeiro FV, Corrêa MG, Franck FC, Benatti BB, Cirano FR (2020). Impact of natural curcumin on the progression of experimental periodontitis in diabetic rats. J Periodont Res.

[B129] Pourbagher-Shahri AM, Farkhondeh T, Ashrafizadeh M, Talebi M, Samargahndian S (2021). Curcumin and cardiovascular diseases: Focus on cellular targets and cascades. Biomed Pharmacother.

[B130] Pradeep A, Garg V, Raju A, Singh P (2016). Adjunctive local delivery of Aloe vera gel in patients with type 2 diabetes and chronic periodontitis: a randomized, controlled clinical trial. J Periodontol.

[B131] Pradeep AR, Suke DK, Martande SS, Singh SP, Nagpal K, Naik SB (2016). Triphala, a new herbal mouthwash for the treatment of gingivitis: A randomized controlled clinical trial. J Periodontol.

[B132] Prasetya RC, Purwanti N, Haniastuti T (2014). Infiltrasi neutrofil pada tikus dengan periodontitis setelah pemberian ekstrak etanolik kulit manggis. Majalah Kedokteran Gigi Indonesia.

[B133] Preshaw PM, Bissett SM (2019). Periodontitis and diabetes. Br Dent J.

[B134] Purohit RN, Bhatt M, Purohit K, Acharya J, Kumar R, Garg R (2017). Clinical and radiological evaluation of turmeric powder as a pulpotomy medicament in primary teeth: An in vivo study. Int J Clin Pediatr.

[B135] Rajeshkumar S, Roy A, Santhoshkumar J, Lakshmi T, Gurunathan D (2019). Antibacterial activity of silver nanoparticles mediated Aloe vera with neem against dental pathogens. Indian J Public Health Res Dev.

[B136] Ramadan DE, Hariyani N, Indrawati R, Ridwan RD, Diyatri I (2020). Cytokines and chemokines in periodontitis. Eur J Dent.

[B137] Raut CP, Sethi KS (2016). Comparative evaluation of co-enzyme Q10 and Melaleuca alternifolia as antioxidant gels in treatment of chronic periodontitis: A clinical study. Contemp Clin Dent.

[B138] Ravishankar PL, Kumar YP, Anila EN, Chakraborty P, Malakar M, Mahalakshmi R (2017). Effect of local application of curcumin and ornidazole gel in chronic periodontitis patients. Int J Pharm Investig.

[B139] Ren M, Zhao Y, He Z, Lin J, Xu C, Liu F (2021). Baicalein inhibits inflammatory response and promotes osteogenic activity in periodontal ligament cells challenged with lipopolysaccharides. BMC Complement Med Ther.

[B140] Rezaee-Tazangi F, Varaa N, Khorsandi L, Abbaspour M (2020). Effects of silymarin-loaded polylactic-co-glycolic acid nanoparticles on osteoarthritis in rats. Iran J Sci Technol Trans A: Sci.

[B141] Rezaei-Tazangi F, Roghani-Shahraki H, Khorsand Ghaffari M, Abolhasani Zadeh F, Boostan A, ArefNezhad R, Motedayyen H (2021). The therapeutic potential of common herbal and nano-based herbal formulations against ovarian cancer: new insight into the current evidence. Pharmaceuticals.

[B142] Sabbaghzadegan S, Golsorkhi H, Soltani MH, Kamalinejad M, Bahrami M, Kabir A, Dadmehr M (2021). Potential protective effects of Aloe vera gel on cardiovascular diseases: A mini‐review. Phytother Res.

[B143] Sahu PK, Giri DD, Singh R, Pandey P, Gupta S, Shrivastava AK, Kumar A, Pandey KD (2013). Therapeutic and medicinal uses of Aloe vera: a review. Pharmacol Pharm.

[B144] Salama MT, Alsughier ZA (2019). Effect of green tea extract mouthwash on salivary streptococcus mutans counts in a group of preschool children: an in vivo study. Int J Clin Pediatr.

[B145] Samadi F, Kahrizi MS, Heydari F, Arefnezhad R, Roghani-Shahraki H, Ardekani AM, Rezaei-Tazangi F (2022). Quercetin and osteoarthritis: A mechanistic review on the present documents. Pharmacology.

[B146] Saxena S, Lakshminarayan N, Gudli S, Kumar M (2017). Anti bacterial efficacy of Terminalia chebula, Terminalia bellirica, Embilica officinalis and Triphala on salivary Streptococcus mutans count–a linear randomized cross over trial. J Clin Diagnostic Res.

[B147] Sayad A, Mirzajani S, Gholami L, Razzaghi P, Ghafouri-Fard S, Taheri M (2020). Emerging role of long non-coding RNAs in the pathogenesis of periodontitis. Biomed Pharmacother.

[B148] Sell AM, de Alencar JB, Visentainer JEL, Silva CDO (2017). Immunopathogenesis of Chronic Periodontitis. Periodontitis.

[B149] Seth TA, Kale TA, Lendhey SS, Bhalerao PV (2022). Comparative evaluation of subgingival irrigation with propolis extract versus chlorhexidine as an adjunct to scaling and root planing for the treatment of chronic periodontitis: A randomized controlled trial. J Indian Soc Periodontol.

[B150] Seymour GJ, Gemmell E, Reinhardt RA, Eastcott J, Taubman MA (1993). Immunopathogenesis of chronic inflammatory periodontal disease: cellular and molecular mechanisms. J Periodontal Res.

[B151] Sezer U, Kara Mİ, Erciyas K, Özdemir H, Üstün K, Özer H, Göze F (2013). Protective effects of ginkgo biloba extract on ligature-induced periodontitis in rats. Acta Odontol Scand.

[B152] Sha AM, Garib BT, Azeez SH, Gul SS (2021). Effects of curcumin gel on osteoclastogenic bone markers in experimental periodontitis and alveolar bone loss in wistar rats. J Dent Sci.

[B153] Shakib Z, Shahraki N, Razavi BM, Hosseinzadeh H (2019). Aloe vera as an herbal medicine in the treatment of metabolic syndrome: A review. Phytother Res.

[B154] Shamim R, Satpathy A, Nayak R, Mohanty R, Panda S (2016). Efficacy of fresh Aloe vera extract in postoperative healing following periodontal surgery in patients with chronic periodontitis: A randomized clinical trial. J Dent Allied Sci.

[B155] Sharma RA, Gescher AJ, Steward WP (2005). Curcumin: the story so far. Eur J Cancer.

[B156] Shi J, Zhang Y, Zhang X, Chen R, Wei J, Hou J, Wang B, Lai H, Huang Y (2021). Remodeling immune microenvironment in periodontitis using resveratrol liposomes as an antibiotic-free therapeutic strategy. J Nanobiotechnology.

[B157] Shi W, Ling D, Zhang F, Fu X, Lai D, Zhang Y (2021). Curcumin promotes osteogenic differentiation of human periodontal ligament stem cells by inducting EGR1 expression. Arch Oral Biol.

[B158] Sigusch B, Klinger G, Glockmann E, Simon HU (1998). Early‐onset and adult periodontitis associated with abnormal cytokine production by activated T lymphocytes. J Periodontol.

[B159] Singh AK, Yadav S, Sharma K, Firdaus Z, Aditi P, Neogi K, Bansal M, Gupta MK, Shanker A, Singh RK, Prakash P (2018). Quantum curcumin mediated inhibition of gingipains and mixed-biofilm of Porphyromonas gingivalis causing chronic periodontitis. RSC Adv.

[B160] Socransky SS, Haffajee AD (2005). Periodontal microbial ecology. Periodontol.

[B161] Songsiripradubboon S, Banlunara W, Sangvanich P, Trairatvorakul C, Thunyakitpisal P (2016). Clinical, radiographic, and histologic analysis of the effects of acemannan used in direct pulp capping of human primary teeth: short-term outcomes. Odontology.

[B162] Soulissa AG, Afifah J, Widyarman AS (2020). The effect of tea tree oil in inhibiting the adhesion of pathogenic periodontal biofilms in vitro. Sci Dent J.

[B163] Souto R, Andrade AFBD, Uzeda M, Colombo APV (2006). Prevalence of" non-oral" pathogenic bacteria in subgingival biofilm of subjects with chronic periodontitis. Braz J Microbiol.

[B164] Souza M, Lopes L, Bonez P, Gündel A, Martinez D, Sagrillo M, Giongo JL, Vaucher RA, Raffin RP, Boligon AA, Santos RC (2017). Melaleuca alternifolia nanoparticles against Candida species biofilms. Microb Pathog.

[B165] Sreedhar A, Sarkar I, Rajan P, Pai J, Malagi S, Kamath V, Barmappa R (2015). Comparative evaluation of the efficacy of curcumin gel with and without photo activation as an adjunct to scaling and root planing in the treatment of chronic periodontitis: A split mouth clinical and microbiological study. J Nat Sci Biol Med.

[B166] Subramani K, Shanmugam BK, Rangaraj S, Palanisamy M, Periasamy P, Venkatachalam R (2018). Screening the UV-blocking and antimicrobial properties of herbal nanoparticles prepared from Aloe vera leaves for textile applications. IET Nanobiotechnol.

[B167] Sun JY, Li DL, Dong Y, Zhu CH, Liu J, Li JD (2016). Baicalin inhibits toll-like receptor 2/4 expression and downstream signaling in rat experimental periodontitis. Int Immunopharmacol.

[B168] Suri SS, Fenniri H, Singh B (2007). Nanotechnology-based drug delivery systems. J Occup Med Toxicol.

[B169] Susanto C, Lokanata S, Ningrum JW (2021). The effect of hydrogel Aloe vera (Aloe vera (L ) burm) on the number of neutrophil cells in aggressive periodontitis induced by aggregatibacter actinomycetemcomitans. J Biomed Transl Res.

[B170] Suzuki J-i, Aoyama N, Ogawa M, Hirata Y, Izumi Y, Nagai R, Isobe M (2010). Periodontitis and cardiovascular diseases. Expert Opin Ther Targets.

[B171] Taalab MR, Mahmoud SA, El Moslemany RM, Abdelaziz DM (2021). Intrapocket application of tea tree oil gel in the treatment of stage 2 periodontitis. BMC Oral Health.

[B172] Taleghani F, Rezvani G, Birjandi M, Valizadeh M (2018). Impact of green tea intake on clinical improvement in chronic periodontitis: a randomized clinical trial. J Stomatol Oral Maxillofac Surg.

[B173] Tankeu S, Vermaak I, Kamatou G, Viljoen A (2014). Vibrational spectroscopy as a rapid quality control method for Melaleuca alternifolia Cheel (tea tree oil). Phytochem Anal.

[B174] Tan L, Cao Z, Chen H, Xie Y, Yu L, Fu C, Zhao W, Wang Y (2021). Curcumin reduces apoptosis and promotes osteogenesis of human periodontal ligament stem cells under oxidative stress in vitro and in vivo. Life Sci.

[B175] Taskan MM, Gevrek F (2020). Quercetin decreased alveolar bone loss and apoptosis in experimentally induced periodontitis model in wistar rats. Antiinflamm Antiallergy Agents Med Chem.

[B176] Terzi V, Morcia C, Faccioli P, Vale G, Tacconi G, Malnati M (2007). In vitro antifungal activity of the tea tree (Melaleuca alternifolia) essential oil and its major components against plant pathogens. Lett Appl Microbiol.

[B177] Theodoro LH, Ferro-Alves ML, Longo M, Nuernberg MAA, Ferreira RP, Andreati A, Ervolino E, Duque C, Garcia VG (2017). Curcumin photodynamic effect in the treatment of the induced periodontitis in rats. Lasers Med Sci.

[B178] Tian M, Chen G, Xu J, Lin Y, Yi Z, Chen X, Li X, Chen S (2022). Epigallocatechin gallate-based nanoparticles with reactive oxygen species scavenging property for effective chronic periodontitis treatment. Chem Eng J.

[B179] Vangipuram S, Jha A, Bhashyam M (2016). Comparative efficacy of aloe vera mouthwash and chlorhexidine on periodontal health: A randomized controlled trial. J Clin Exp Dent.

[B180] Venugopal P, Koshy T, Lavu V, Ranga Rao S, Ramasamy S, Hariharan S, Venkatesan V (2018). Differential expression of microRNAs let‐7a, miR‐125b, miR‐100, and miR‐21 and interaction with NF‐kB pathway genes in periodontitis pathogenesis. J Cell Physiol.

[B182] Wang ZL, Wang S, Kuang Y, Hu Z M, Qiao X, Ye M (2018). A comprehensive review on phytochemistry pharmacology and flavonoid biosynthesis of Scutellaria baicalensis. Pharm Biol.

[B183] Wang W, Xi M, Duan X, Wang Y, Kong F (2015). Delivery of baicalein and paclitaxel using self-assembled nanoparticles: synergistic antitumor effect in vitro and in vivo. Int J Nanomed.

[B184] Wang Y, Andrukhov O, Rausch-Fan X (2017). Oxidative stress and antioxidant system in periodontitis. Front Physiol.

[B185] Wang Y, Li C, Wan Y, Qi M, Chen Q, Sun Y (2021). Quercetin‐loaded ceria nanocomposite potentiate dual‐directional immunoregulation via macrophage polarization against periodontal inflammation. Small.

[B186] Warad SB, Kolar SS, Kalburgi V, Kalburgi NB (2013). Lemongrass essential oil gel as a local drug delivery agent for the treatment of periodontitis. Anc Sci life.

[B187] Wei Y, Fu J, Wu W, Ma P, Ren L, Yi Z, Wu J (2021). Quercetin prevents oxidative stress-induced injury of periodontal ligament cells and alveolar bone loss in periodontitis. Drug Des Devel Ther.

[B188] Wilensky A, Chaushu S, Shapira L (2015). The role of natural killer cells in periodontitis. Periodontol.

[B189] Xiong Y, Zhao B, Zhang W, Jia L, Zhang Y, Xu X (2020). Curcumin promotes osteogenic differentiation of periodontal ligament stem cells through the PI3K/AKT/Nrf2 signaling pathway. Iran J Basic Med Sci.

[B190] Yadav E, Kumar S, Mahant S, Khatkar S, Rao R (2017). Tea tree oil: a promising essential oil. J Essent Oil Res.

[B191] Yadav D, Kumar A, Kumar P, Mishra D (2015). Antimicrobial properties of black grape (Vitis vinifera ) peel extracts against antibiotic-resistant pathogenic bacteria and toxin producing molds. Indian J pharmacol.

[B192] Yamaguchi M, Weitzmann MN (2011). Zinc stimulates osteoblastogenesis and suppresses osteoclastogenesis by antagonizing NF-κB activation. Mol Cell Biochem.

[B193] Yang X, Huang B, Chen J, Huang S, Zheng H, Lun Z-R, Shen J, Wang Y, Lu F (2012). In vitro effects of aqueous extracts of Astragalus membranaceus and Scutellaria baicalensis GEORGI on Toxoplasma gondii. Parasitol Res.

[B194] Ye F, Che Y, McMillen E, Gorski J, Brodman D, Saw D, Jiang B, Zhang D Y (2009). The effect of Scutellaria baicalensis on the signaling network in hepatocellular carcinoma cells. Nutr Cancer.

[B195] Xiao C-J, Yu X-J, Xie J-L, Liu S, Li S (2018). Protective effect and related mechanisms of curcumin in rat experimental periodontitis. Head Face Med.

[B196] Xing J, Chen X, Zhong D (2005). Absorption and enterohepatic circulation of baicalin in rats. Life Sci.

[B197] Zacarias JMV, de Alencar JB, Tsuneto PY, Souza VHD, Silva CO, Visentainer JEL, Sell AM (2019). The influence of TLR4, CD14, OPG, and RANKL polymorphisms in periodontitis: A case-control study. Mediators Inflamm.

[B198] Zambrano LM, Brandao DA, Rocha FR, Marsiglio RP, Longo IB, Primo FL, Tedesco AC, Guimaraes-Stabili MR, Rossa Junior C (2018). Local administration of curcumin-loaded nanoparticles effectively inhibits inflammation and bone resorption associated with experimental periodontal disease. Sci Rep.

[B199] Zanuzzo FS, Sabioni RE, Montoya LNF, Favero G, Urbinati EC (2017). Aloe vera enhances the innate immune response of pacu (Piaractus mesopotamicus) after transport stress and combined heat killed Aeromonas hydrophila infection. Fish Shellfish Immunol.

[B200] Zhao M-X, Zhu B-J (2016). The research and applications of quantum dots as nano-carriers for targeted drug delivery and cancer therapy. Nanoscale Res Lett.

[B201] Zhao T, Tang H, Xie L, Zheng Y, Ma Z, Sun Q, Li X (2019). Scutellaria baicalensis Georgi (Lamiaceae): a review of its traditional uses, botany, phytochemistry, pharmacology and toxicology. J Pharm Pharmacol.

[B202] Zhen L, Fan D-s, Zhang Y, Cao X-m, Wang L-m (2015). Resveratrol ameliorates experimental periodontitis in diabetic mice through negative regulation of TLR4 signaling. Acta Pharmacol Sin.

[B203] Zhou T, Chen D, Li Q, Sun X, Song Y, Wang C (2013). Curcumin inhibits inflammatory response and bone loss during experimental periodontitis in rats. Acta Odontol Scand.

